# Safety, pharmacokinetics, and pharmacodynamics of Dexmedetomidine two-phase flow atomization in healthy subjects: a randomized, parallel study

**DOI:** 10.1186/s12871-025-03358-7

**Published:** 2025-10-01

**Authors:** Yujun Lian, Huanqi Luo, Zhijing Yang, Jiadi He, Haozhu Chen, Yanyan Sun

**Affiliations:** Department of Anesthesiology, Shenzhen University General Hospital, 1298 Xueyuan Avenue, Nanshan District, Shenzhen, Guangdong 518055 China

**Keywords:** Dexmedetomidine (DEX), Two-phase flow atomization, Nasal drug delivery, Pharmacokinetics

## Abstract

**Background:**

In this study, we aimed to compare the sedative effects, pharmacokinetics (PKs), and safety of dexmedetomidine (DEX) administered using a two-phase flow atomization device with those of conventional intranasal drop-based administration in healthy volunteers.

**Methods:**

This prospective, parallel, double-blind study compared the PK and pharmacodynamic (PD) profiles of DEX administered via conventional intranasal drop (Group C) or two-phase flow atomization (Group E). Twenty-two healthy adult volunteers were enrolled and randomly assigned to the two groups using a computer-generated randomization sequence. Each participant received DEX at a dose of 2 µg/kg. Sedation was assessed using the Ramsay Sedation Scale and the bispectral index, and PK analysis was performed on blood samples collected at multiple time points.

**Results:**

The two-phase flow atomization group exhibited a significantly faster onset of sedation than the conventional intranasal drop-based administration group and a longer duration of sedation. Compared with Group C, Group E presented an increasing trend in the maximum plasma concentration and in the area under the concentration‒time curve from time zero to the last time point (AUC0t). We found no significant differences in other PK parameters between the two groups, with all *P*-values > 0.05. The plasma concentrations were slightly higher in the two-phase flow atomization group after 30 min. The changes in systolic blood pressure and heart rate of both groups of volunteers were within 25% of the baseline values, with no adverse reactions such as hypotension or sinus bradycardia.

**Conclusions:**

The findings indicate that the two-phase flow atomization device offers faster onset and longer duration of sedation with DEX nasal administration, with a favorable safety profile in healthy volunteers (American Society of Anesthesiologists Physical Status Class 1) under specific conditions. Therefore, based on the sedation dynamics, we infer that this device may enhance drug deposition in the olfactory region of the nasal cavity, thereby enhancing the effects of the drug in the central nervous system through nose-to-brain delivery. This study provides new insights into the optimization of nasal drug delivery devices, particularly for pediatric patients and patients in clinical settings requiring rapid sedation.

**Trial registration:**

This study was registered at ChiCTR.org.cn (registration number ChiCTR2400091480) on 29/10/2024.

**Supplementary Information:**

The online version contains supplementary material available at 10.1186/s12871-025-03358-7.

## Background

Dexmedetomidine (DEX) is a highly selective α_2_-adrenergic receptor agonist that acts on α_2_ receptors in the locus coeruleus to induce a hypnotic effect, resulting in a state of unconsciousness similar to natural sleep. Its uniqueness lies in its ability to maintain patients in an easily arousable and cooperative state, with minimal respiratory depression and only mild hemodynamic effects. In recent years, the United States Food and Drug Administration has approved DEX for procedural sedation, and its clinical applications have gradually expanded to include sedation in pediatric patients, intranasal and oral administration, and use as an adjunct to local analgesia [1–3]. Intranasal administration provides high bioavailability, involves minimal procedural risk, and allows for noninvasive self-administration, significantly improving cost-effectiveness and patient compliance [4, 5]. A large body of clinical data supports the efficacy of intranasal DEX in both pediatric and adult patients [6].

Conventional intranasal delivery modalities, such as nasal droppers and sprays, are operationally straightforward, but they exhibit suboptimal drug deposition efficiency. Anatomical constraints of the nasal cavity, such as its narrow lumen, turbulent airflow patterns, and mucociliary clearance mechanisms, result in less than 5% of administered drug doses reaching the olfactory region, a critical site for nose-to-brain transport [7, 8]. Consequently, a substantial proportion of drug particles are either deposited in the nasal vestibule (≥ 50%) or inadvertently aspirated into the lower respiratory tract [9]. The narrow structure and complex internal anatomy of the nasal cavity cause large drug particles to become deposited in the nasal vestibule and small particles to enter the lungs directly, resulting in suboptimal deposition in the effective absorption area of the nasal cavity [7]. Recent studies have shown that nebulized drug delivery provides several advantages, as nebulizers producing uniform particles at a lower velocity, increasing deposition in target areas such as the middle and superior turbinates [10–12]. However, Li et al. [13] reported no significant differences in pharmacodynamics (PDs), pharmacokinetics (PKs), or sedation effects between intranasal DEX delivered via a 1-mL syringe and a nebulizer (MAD Nasal™, Wolfe Tory Medical, Inc., Salt Lake City, UT, USA). Similarly, B.L. Li et al. [14] reported no significant difference in sedation success rates between nebulized and nasal DEX administration in pediatric patients undergoing transthoracic echocardiography. In terms of whether these discrepancies are related to the type of nebulizer used, Vecellio et al. [15] compared the deposition rates of two commonly used nebulizers (Atomisor NL11S^®^ sonic, DTF-Medical, France; Aeroneb Solo^®^, Aerogen, Galway, Ireland; DTF-Aerodrug, Tours, France) in the nasal cavity and reported deposition rates of 9% and 27%, respectively, with 33–56% of the drug being deposited in the lungs [16]. The olfactory region, a key absorption area for nose-to-brain drug delivery, has a deposition rate of only 0.5–5% with conventional nasal sprays and nebulizers [7]. Therefore, the efficiency of nebulized drug delivery may be a critical factor affecting drug efficacy.

The complexity and interspecies anatomical differences in nasal structures render most animal models unsuitable for studying intranasal nebulized drug deposition in humans. In vitro simulation experiments were previously conducted to compare the effects of different nebulization parameters and delivery modes on nasal and olfactory deposition rates. We developed an improved two-phase flow intranasal drug delivery device (patent application number: 202310793138.0) and demonstrated that this device significantly improved the nasal and olfactory deposition rates, with the nasal and olfactory deposition rates reaching 70% and 15%, respectively.

In this study, we aimed to compare the sedation effects, adverse reactions, and plasma concentrations of DEX delivered via two-phase flow nebulization with those of conventional intranasal drop-based delivery in healthy volunteers to validate the efficacy and safety of this device.

## Methods

**Fig. 1:**
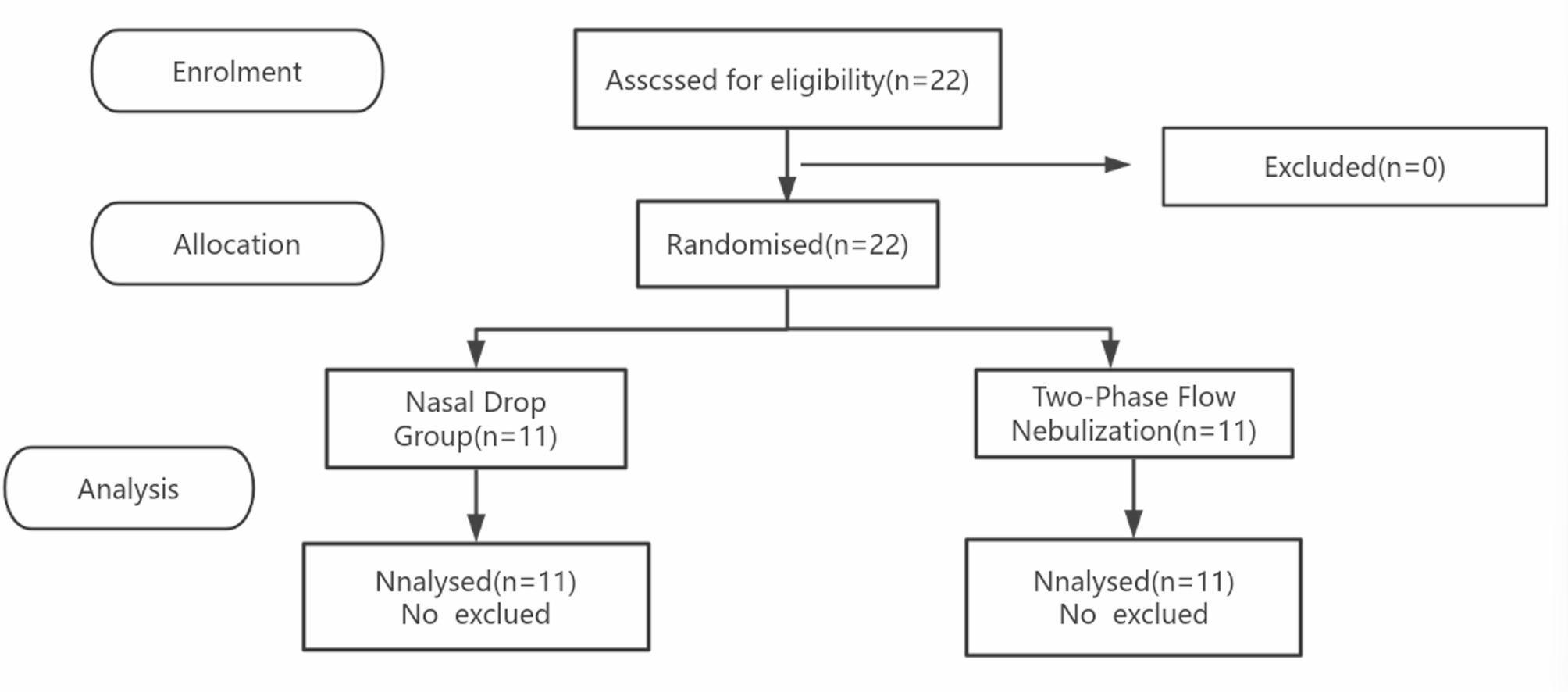
Flow diagram of patient enrollment, allocation, and analysis

The study protocol was approved by the Ethics Committee of Shenzhen University General Hospital (SUGHKYLL-2021-07-29-1) and registered with the Chinese Clinical Trial Registry (ChiCTR2400091480). Twenty-two healthy adult volunteers aged 18–45 years were recruited for this randomized, double-blind preclinical study. Eligible participants were healthy adults with an American Society of Anesthesiologists (ASA) physical status class of I. The exclusion criteria were as follows: body mass index >30 kg/m^2^; history of intolerance to the study drug or related compounds; concomitant drug therapy of any kind, except paracetamol within 14 days prior to the study; current or past history of alcoholism, drug abuse, or cigarette smoking; or abnormal electrocardiogram findings. An independent investigator randomly divided the participants into the conventional intranasal drop-based administration group (Group C) and the two-phase flow atomization group (Group E) using computer-generated randomization software. One operator administered the drug, and three blinded observers recorded the data and collected blood samples. All blood samples were recorded before analysis (Fig.1).

After entering the room, a 20G peripheral intravenous catheter was placed in the upper limb of the volunteers for blood sampling and, if necessary, fluid therapy. The heart rate (HR), noninvasive systolic blood pressure (SBP), diastolic blood pressure (DBP), oxygen saturation (SpO_2_), and bispectral index (BIS) were monitored. A 2 µg/kg dose of undiluted DEX (supplied by Jiangsu Hengrui) was then administered. For the nasal drop group, the undiluted DEX at 2 µg/kg was drawn into 1-mL tuberculin syringes, and the drug was evenly distributed between both nostrils. For the atomization group, the dead space of the atomizer was filled with DEX prior to administration. This atomizer delivers 0.1 mL per actuation with a 10-s nebulization time. All volunteers were placed in the supine position, and the operator alternately administered 0.1 mL of the drug into each nostril via a 1-mL syringe or a nebulization device; this was repeated every 2–3 min until the entire dose was delivered, for a total duration of 5–10 min. The sedation levels were assessed using the Ramsay Sedation Scale and BIS monitoring. Sedation scores and blood samples were collected at baseline and at 5, 10, 15, 20, 30, 45, 60, 90, 120, 180, and 240 min post-administration (up to 4 h). The HR, BP, SpO2, and BIS values and adverse reactions were recorded. The plasma was separated by centrifugation (1,500 rcf, 4 °C, within 10 min of collection). The upper plasma layer (2 mL) was placed in anticoagulant tubes, labeled according to a randomization table, and stored at 4 °C. All plasma samples were sent to the Shenzhen Shenjian Medical Laboratory for drug concentration analysis. The detection method involved placing 200 µL of each plasma sample in a 2-mL centrifuge tube, followed by adding 50 µL of carbamazepine solution (10 ng/mL) or 100 µL of 0.2 mol/L sodium hydroxide solution. The mixture was vortexed for 1 min, followed by the addition of 1 mL of ethyl acetate-dichloromethane (4:1). After sealing and vortexing for 2 min, the mixture was centrifuged (20,000 rcf, 4 °C, for 15 min). Next, 800 µL of the organic layer was transferred to a new 2-mL centrifuge tube, dried under nitrogen, and reconstituted in 50 µL of methanol-water (70:30). Subsequently, the mixture was centrifuged (20,000 rcf, 4 °C, 15 min), after which the mixture was used for Liquid Chromatography‒Tandem Mass Spectrometry (LC‒MS/MS) analysis. The LC–MS analysis conditions were as follows: column, Agilent Eclipse Plus C18 (2.1 × 100 mm, 2.7 μm); column temperature, 40 °C; injection volume, 5 µL; mobile phase: A = 0.1% formic acid in water; and B = methanol. Finally, through comparison with the standard methodology, the specific information of the standard is shown in Table 1. The DEX plasma concentration was determined by qualitative analysis based on retention time and quantitative analysis based on the peak area.

**Table 1:** Standard curve information

No	Name	Retention Time (min)	Linear range (ng/mL)	Linear Equation	*r*	LOQ(ng/mL)	LOD(ng/mL)
1	Dexmedetomidine	2.11	0.100 ~ 10.0	y = 0.05166x + 0.00181	0.9997	0.0250	0.00750

### Pharmacodynamic indicators

Onset of sedation refers to the time interval between drug administration and the first occurrence of a BIS ≤ 85 and a Ramsay score ≥ 3. Duration of sedation refers to the cumulative time during which both a BIS ≤ 85 and Ramsay score ≥ 3 are simultaneously maintained.

## Pharmacokinetic indicators

The PK parameters of DEX were analyzed using the noncompartmental model in DAS 2.0. The main PK parameters included maximum plasma concentration (Cmax), area under the concentration‒time curve from 0 to the last time point (AUC0-t), time to reach Cmax (Tmax), terminal elimination half-life (t1/2), apparent volume of distribution corrected for bioavailability (V/F), clearance corrected for bioavailability (CL/F), and mean residence time from 0 to the last time point (MRT0-t).

### Statistical analysis

All continuous variables were normalized. Continuous variables are expressed as the mean ± standard deviation (normal distribution) or as the median (interquartile range [IQR]: 25–75%) (nonnormal distribution). Comparisons were made using independent t-tests or rank-sum tests. Categorical variables were expressed as percentages and compared using the chi-square test. Statistical analysis was performed using the Statistical Package for the Social Sciences for Windows version 13.0 (SPSS Inc., Chicago, IL, USA). A *P*-value < 0.05 was considered statistically significant.

## Results

### Demographics

All 22 volunteers completed the study (*n* = 11 per group). Table 2 shows the demographic data of the volunteers. The average age of the participants in Group C was 31.45 ± 5.15 years, and 33.73 ± 7.39 years in Group E. The male-to-female ratio was 72.73%:27.27% in Group C and 63.64%:36.36% in Group E. The mean body weight was 64.23 ± 11.25 kg in Group C and 60.14 ± 12.32 kg in Group E. The mean pre-administration BIS value was 96.64 ± 1.69 in Group C and 96.45 ± 0.82 in Group E. The mean pre-administration HR was 69.82 ± 7.29 bpm in Group C and 64.83 ± 7.52 bpm in Group E. The mean pre-administration SBP was 113.55 ± 11.25 mmHg in Group C and 113.55 ± 10.60 mmHg in Group E. There were no significant differences in the baseline data between the two groups (*P* > 0.05, Table 2) .

**Table 2:** Demographics and clinical characteristics of the control and experimental groups

Parameters	Group C (nasal drops) (*n* = 11)	Group E (two-phase Atomization) (*n* = 11)	t/χ^2^	*P*
Age(years)	31.45 ± 5.15	33.73 ± 7.39	−0.837	0.410
Gender(%)			−0.210	0.650
Men	8 (72.73)	7(63.64)		
Women	3(27.27)	4(36.36)		
Weight(kg)	64.23 ± 11.25	69.14 ± 12.32	−0.976	0.340
BIS	96.64 ± 1.69	96.45 ± 0.82	0.321	0.750
HR(bpm)	69.82 ± 7.29	64.83 ± 7.52	1.580	0.129
SBP (mmHg)	113.55 ± 11.25	113.55 ± 10.60	0.000	1.000

Data are presented as the means ± standard deviations, except for sex (male/female). *F* Female, *M* Male, *SD* standard deviation, *BIS* Bispectral index, *HR* Heart rate, *SBP* Systolic blood pressure

### Hemodynamics

**Fig. 2:**
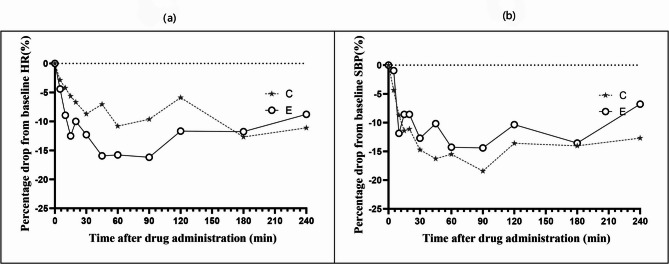
(**a**) Mean percentage change in HR from baseline. (**b**) Mean percentage of SBP from baseline. C: Nasal drop group, shown as a star with a long dashed line. E: Two-phase atomization group, shown as a circle with a solid line

The changes in HR and SBP from baseline in both groups are shown in Fig. 2. Group E showed a more significant decrease in HR than Group C, whereas Group C showed a more significant decrease in SBP than Group E. However, in either group, the values did not drop by more than 25% from baseline, and no instances of sinus bradycardia or hypotension were observed.

## Pharmacodynamic evaluation

Table 3 presents the statistical analysis of the original data regarding the onset and duration of sedation. The median onset time of Ramsay sedation was 10 min (IQR: 10–20 min) in Group C and 5 min (IQR: 3–10 min) in Group E (Table 3). The onset time of BIS sedation was 16 min (IQR: 15–20 min) in Group C and 5 min (IQR: 3–13 min) in Group B (Table 3). Therefore, regardless of whether the Ramsay score or the BIS was used as the evaluation standard, the two-phase flow device administration resulted in a significantly faster onset of sedation (*P* < 0.05).

**Table 3:** Rank sum test of sedation onset time and duration for the two administration routes. Values are presented as the median (interquartile range)

		Group C (nasal drops) (*n* = 11)	Group E (two-phase Atomization) (*n* = 11)	Z	*P*
Ramsay Onset time (min)	10 (10–20)		5 (3–10)	−2.709	0.007
BIS Onset time (min)	16 (15–20)		5 (3–13)	−2.957	0.003
Ramsay Sedation duration (min)	170 (60–225)		225 (175–235)	−2.017	0.004
BIS Sedation duration (min)	80 (15–225)		225 (175–230)	−2.080	0.038

**Fig. 3:**
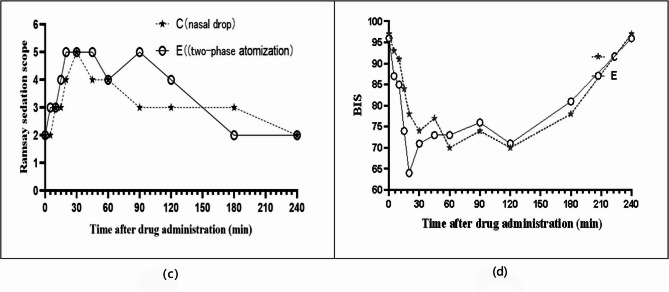
(**c**) Median Ramsay sedation score and (**d**) median BIS sedation duration. C: Nasal drop group, shown as a star with a long dashed line. E: Two-phase atomization group, shown as a circle with a solid line

Similarly, the median maintenance times of Ramsay sedation for nasal drops and two-phase flow device administration were 170 min (IQR: 60–225 min) and 225 min (IQR: 175–235 min), respectively (Table 3). The duration of BIS sedation was 80 min (IQR: 15–225) in Group C and 225 min (IQR: 175–230) in Group E (Table 3). These results revealed that two-phase flow device administration significantly increased the maintenance time of sedation (*P* < 0.05). Figure 3 shows the average Ramsay scores and BIS values at each time point for the volunteers in both groups. Group E exhibited more pronounced changes in Ramsay and BIS values during the early stages post-administration, suggesting that the volunteers in Group E reached a sedative state faster at the same dosage. Moreover, the highest or lowest average Ramsay scores and BIS values were observed in Group E volunteers, indicating that Group E could achieved a deeper level of sedation at the same dosage.

## Pharmacokinetic evaluations

**Fig. 4:**
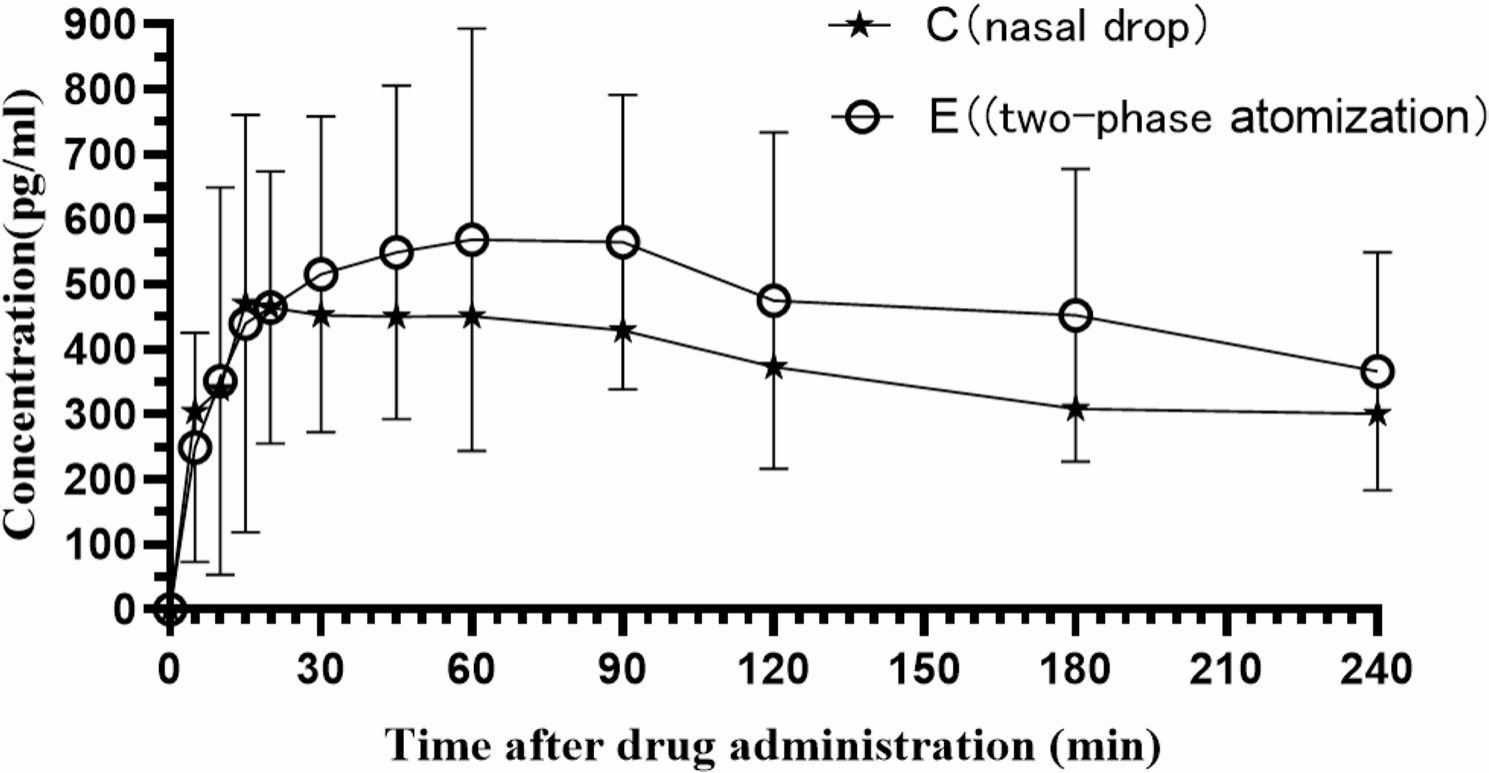
Mean concentration–time curves of DEX in healthy subjects following intranasal administration of 2 µg/kg via spray or drops

The average concentration‒time curves of DEX for both groups are shown in Fig. 4, and the PK parameters of DEX for the two groups are listed in Table 4.

**Table 4:** Pharmacokinetic properties of Dexmedetomidine (DEX) in healthy subjects after a single intranasal dose of DEX via spray or drops, calculated using DAS2.0.3 T test of all the PK, values are presented as the mean ± sd

PK paramete	Group C (nasal drops)	Group E (two-phase Atomization)	t	*P*
Cmax (pg/ml)	573.10 ± 323.02	701.82 ± 262.33	−1.026	0.317
AUC0-t (pg*min/mL)	110.74± 71.18	112.63± 50.97	−0.072	0.944
Tmax (min)	61.36 ± 68.99	60.45 ± 46.98	0.036	0.972
t1/2z(min)	288.85 ± 197.20	226.22 ± 100.72	0.938	0.359
Vz/F(ml/kg)	41492.99 ± 17277.46	37333.26 ± 38410.69	0.328	0.747
CLz/F(ml/min/kg)	145.73 ± 106.38	118.71 ± 90.07	0.643	0.528
MRT(0-t)min	112.41 ± 7.97	116.25 ± 5.87	−1.285	0.213

Values are expressed as the means ± SDs

*Abbreviations*: *PK* Pharmacokinetics, *Cmax* Maximum plasma concentration, *AUC0-t* Area under the concentration‒time curve from time zero to the last time point

As shown in Fig. 4, at the same dosage of 2 µg/kg, the mean plasma concentrations of Groups E and C were nearly identical within the first 24 min post-administration. After 30 min, the concentration in Group E was greater than that in Group C.

Compared with Group C, Group E presented an increasing trend in the maximum plasma concentration (Cmax) (701.82 ± 262.33 pg/mL vs. 573.10 ± 323.02 pg/mL) and in the AUC0t (112.63 ± 50.97 pg*min/mL vs. 110.74 ± 71.18 pg*min/mL). However, there were no statistically significant differences compared with Group C, and no significant differences in other PK parameters between the two groups, with all P values > 0.05 (Tmax: 61.36 ± 68.99 min vs. 60.45 ± 46.98 min; T1/2z: 288.85 ± 197.20 min vs. 26.22 ± 100.72 min; Vz/F: 4142.99 ± 17277.46 mL/kg vs. 37333.26 ± 38410.69 mL/kg; CLz/F: 145.73 ± 106.38 mL/min/kg vs.118.71 ± 90.07 mL/min/kg; MRT(0-t): 112.41 ± 7.97 min vs.116.25 ± 5.87 min).

## Discussion

From a PD perspective, our results (Table 3; Fig. 3) showed that Group E achieved a faster onset, longer duration, and deeper level of sedation than Group C, irrespective of whether the Ramsay score or BIS was used as the evaluation standard. The PK parameters between the two groups showed no significant differences, which contrasted with the findings of Li et al., but aligned with our in vitro simulation results using computational fluid dynamics (CFD) digital modeling technology. According to the final aerosolization parameters, we primarily improved the drug’s olfactory region deposition rate, whereas the deposition rate in other nasal regions (which relied on nasal mucosa-systemic absorption) did not increase significantly. There was no notable difference in PKs parameters related to blood circulation between the two groups. However, when comparing the sedative PDs, the two-phase flow group showed a significant improvement. Based on this, we speculate that this may be attributed to our use of CFD simulation to optimize the nebulization parameters, thereby improving olfactory deposition rates. Since DEX exerts hypnotic effects through α_2_-receptors in the locus coeruleus, improved olfactory deposition may enhance sedation not only through systemic absorption but also through a direct intranasal-to-brain delivery. This is particularly evident in Fig. 4, where the plasma concentration curves of both groups overlap within the first 10–20 min post-administration. Nevertheless, Group E achieved a faster onset of sedation, suggesting that DEX could directly act on the brain through olfactory absorption, leading to deeper sedation despite similar plasma concentrations.

In recent years, the nose-to-brain delivery pathway, which enables drugs to bypass the blood‒brain barrier (BBB) and reach the central nervous system (CNS), has garnered considerable attention [17]. This is largely attributed to the olfactory region, which provides a direct connection between the external environment and the CNS through the olfactory nerve. The BBB is a major impediment to the entry of exogenous compounds into the brain. Intranasal administration facilitates direct brain targeting, circumventing enzymatic and chemical degradation and first-pass hepatic metabolism. The nasal mucosa is richly vascularized and possesses high permeability, allowing for rapid drug absorption [18]. Therefore, intranasal-to-brain delivery holds great promise for the delivery of conventional drugs aimed at treating CNS disorders such as Parkinson’s disease and Alzheimer’s disease, and research in this area is growing. However, research on the intranasal delivery of anesthetic drugs, which require action on the brain, is limited. Traditional intranasal delivery devices, such as droppers, nasal sprays, and nebulizers, struggle to deliver drugs to the olfactory region because of the complex anatomy of the nasal cavity, interindividual variability, and dynamic nasal airflow [19, 20]. These factors present a significant challenge for anesthesiologists, and efforts are currently underway to improve and develop more efficient nebulization methods and devices that can enhance olfactory region targeting for intranasal administration.

CFD is an integration of modern fluid mechanics, numerical mathematics, and computer science. Compared with other fluid dynamics analysis methods, CFD offers significant advantages in terms of cost-effectiveness and time efficiency, while also enabling experimental simulation and analysis of highly complex systems or conditions. These benefits have led to considerable interest in its medical application. In recent years, extensive research has focused on investigating the impact of the geometric parameters of the human respiratory tract on pressure, velocity, and airflow characteristics to enable the use of digital simulation technology in predicting the respiratory system’s flow behavior and performance.

Manniello et al. [21] used computed tomography (CT) scan data from healthy volunteers to create seven adult respiratory tract models and performed CFD simulations with varying particle diameters and velocities. They reported that particles with diameters ranging from 20 to 30 μm and lower velocities achieved higher olfactory deposition rates. Similarly, Ren et al. [22] created a 3D nasal model based on CT data and applied CFD-Discrete Phase Model (DPM) simulations. They reported that a delivery velocity of 15 m/s was more effective for intranasal drug delivery. They also reported that a spray angle of 40° was more suitable for lower auxiliary airflow, whereas a spray angle of 60° was more effective for higher auxiliary airflow. Kleven et al. [23] described how the Norwegian company OptiNose AS used CFD simulations to develop a bidirectional delivery system, which increased olfactory deposition for particles sized 14–18 μm compared to traditional inhalation methods. Although discrepancies exist among the results from different regions, these results fall within reasonable ranges and demonstrate that CT-based respiratory models and CFD simulations can yield reliable results [21, 23, 24]. In China, few reports exist on such research. In collaboration with the Shenzhen McWell Company, we conducted CFD simulations using CT data from six healthy adult Chinese volunteers. The optimal nebulization parameters for a two-phase flow atomization device were identified as follows: particle D50 = 24.32 ± 0.93 μm, mean jet velocity = 0.6 mL/min, mean jet force = 7.2 mN, and mean jet angle = 24°. Under these parameters, the simulated olfactory deposition rate was 10–15%, which was significantly greater than that of traditional nebulizers (≤ 5%) [21].

Consistent with prior studies, the primary safety concerns associated with intranasal DEX are hemodynamic alterations—notably hypotension and bradycardia. However, both groups demonstrated hemodynamic stability, with mean arterial pressure and HR fluctuations remaining within 25% of the baseline values (Fig. 2). Notably, no clinically significant bradycardia or hypotension was observed, supporting the favorable safety profile of the two-phase flow device at the tested dosage (2 µg/kg) [9]. The average blood pressure HR in both groups did not decrease by more than 25% from baseline, and no cases of hypotension or bradycardia were observed. Follow-up 1 day post-procedure revealed no serious adverse events in either group. Therefore, intranasal administration of 2 µg/kg DEX via the two-phase flow device is safe and well-controlled.

In terms of limitations, this study did not include pediatric patients. The clinical application of DEX via nasal administration is relatively well-established in both pediatric and adult patients. Therefore, by combining the results of this study with clinical experience in nasal administration for adults, clinicians can infer the dosage and method of administering DEX through a two-phase flow atomization for pediatric patients. In the future, we plan to include pediatric patients or volunteers in further clinical trials to provide new reference methods for easier management of anesthesia in pediatric patients.

As a major limitation, the study was conducted exclusively in healthy volunteers (ASA 1) under specific conditions, which could limit the generalizability of the results. Additionally, potential variability in nasal anatomy may affect drug absorption. The controlled experimental setting may not fully replicate the clinical conditions. Therefore, future studies involving diverse patient populations are necessary.

Meanwhile, we have only inferred from the PDs of sedation that the drug acts through the nasal-brain pathway, and cannot directly measure CSF to draw conclusions. In the future, we plan to conduct further experiments in animal studies to verify our hypothesis by measuring the drug concentration in the cerebrospinal fluid of the animals.

## Conclusions

The findings indicate that the two-phase flow atomization device offers faster onset and longer duration of sedation with DEX nasal administration, with a favorable safety profile in healthy volunteers (ASA 1) under specific conditions. Therefore, based on the sedation dynamics, we infer that this device may enhance drug deposition in the olfactory region of the nasal cavity, thus enhancing the CNS effects of the drug through the nose-to-brain delivery pathway. This study provides new insights into the optimization of nasal drug delivery devices, particularly for pediatric patients and patients in clinical settings requiring rapid sedation.

## Supplementary Information

Supplementary Material 1. CONSORT guidelines. The study adheres to the CONSORT guidelines. A completed CONSORT checklist has been provided as an additional file.

## Data Availability

The datasets analysed during the current study are available from the corresponding author on reasonable request.

## Glossary

AUC0-t: Area under the concentration–time curve from time zero to the last time point (%)
BBB: Blood-brain barrier
BIS: Bispectral index
BPM: Beat per minute
BMI: Body mass index
CFD: Computational Fluid Dynamics
CFD-DPM: Computational Fluid Dynamics-Discrete Particle Model
Cmax: Maximum plasma concentration'
CNS: Central nervous system
Ct: Computed tomography
DBP: Diastolic blood pressure
DEX: Dexmedetomidine
HR: Heart rate
LC-MS/MS: Liquid chromatography with tandem mass spectrometry
LOD: Limit of detection
LOQ: Limit of quantitation
IQR: Interquartile range
PK: Pharmacokinetics
